# A multivariate prediction model and its application in forecasting acute ischemic stroke: Protocol for a retrospective clinical study

**DOI:** 10.1097/MD.0000000000031695

**Published:** 2022-12-16

**Authors:** Dongmei Yang, Xia Liu, Hui Lan, Li Wang, Xiao Ma, Yu Xie, Jielian Li

**Affiliations:** a Department of Clinical Laboratory, Zigong Third People’s Hospital, Zigong City, China; b Department of Neurology, Zigong Third People’s Hospital, Zigong City, China; c Big Data Research Center, University of Electronic Science and Technology, China; d Medical Examination Center, Zigong Fourth People’s Hospital, Zigong City, China.

**Keywords:** acute ischemic stroke, blood biomarkers, multivariate, prediction model, retrospective study

## Abstract

Acute ischemic stroke (AIS) occurs due to brain ischemia as a result of thrombosis of a cerebral blood vessel. It is a common cerebral blood circulation disorder worldwide and an important cause of death and disability.

**Objective:**

This study aims to establish a prediction model of multiple single category indicators and a joint model, through which to plot multiple receiver operating characteristic curves and compare area under curve of the models so as to predict the occurrence of AIS, explore the pathogenesis of AIS, and provide reference for clinical diagnosis and treatment of AIS.

**Methods:**

A retrospective clinical study was conducted in a Level A tertiary hospital in Sichuan Province, China. The patients participated in this study were over 18 years of age and suffered from acute ischemic stroke. They were hospitalized in department of neurology from October 1, 2019 to September 30, 2022, and underwent coronary artery computed tomographic arteriography (CTA) and blood biomarker detection. We collected demographic information, CTA data and blood biomarker detection values of all these patients.

**Conclusion:**

Through analyzing the clinical data of high-risk groups, this study provides guidance for the prevention and treatment of AIS, and promote further research.

## 1. Introduction

Stroke as a major cause of death, accounts for 10% of the world’s deaths annually. More than 75% of stroke deaths and 81% of disability-adjusted life years (DALYs) occur in low and middle-income countries.^[1]^ In China, the lifetime risk of stroke among people over 20 years of age is 39.9%, ranking first in the world and 25% higher than the world average.^[2]^ According to the basic pathological manifestations, strokes can be classified into 2 categories: hemorrhagic and ischemic. Acute ischemic stroke (AIS) is caused by cerebral ischemia as a result of cerebrovascular thrombosis, accounting for 87% of all stroke types.^[2,3]^ As a common nervous system disease in clinic, acute ischemic stroke has a rapid onset, rapid progress, high disability rate and mortality rate. Early diagnosis and targeted treatment are important methods to control disease progression and improve prognosis.^[4]^

Studies have shown that ischemic stroke is affected by many risk factors, including age, weight, medical history (hypertension, diabetes, hyperlipidemia, coronary heart disease, and atrial fibrillation, etc.), intracranial and extracranial artery plaques, coronary artery stenosis, blood biomarker (total cholesterol, low-density lipoprotein, high-density lipoprotein, triglycerides, and ischemia modified albumin, etc.).^[5–7]^ At present, digital subtraction angiography (DSA) is a reference standard for the diagnosis of vascular diseases in AIS patients. It is a high-risk and invasive operation among critically ill patients, since it may cause temporary or permanent neurological impairment. In clinical practice, computed tomographic arteriography (CTA) which could accurately locate arterial stenosis, occlusion site and occlusion,^[8]^ provides DSA with a low-risk, low-cost and easily available alternative method to detect potential structural vascular abnormalities in a noninvasive manner, predict the risk of hematoma growth and formulate treatment plans. At the same time, the pathogenesis of ischemic stroke is a pathological biochemical reaction process involving a series of blood biochemical markers. At different stages of the occurrence and development of acute ischemic stroke, patients’ blood biochemical indicators will change regularly with pathological changes.^[9]^ Many serum markers closely related to the development of ischemic stroke and disease evaluation can be used as important indicators for early diagnosis, treatment and evaluation of acute ischemic stroke.^[10]^ As the early prevention of AIS has become a top priority of clinical research, clarifying the relationship between acute ischemic stroke and serological markers can provide reference for early prevention and late treatment of acute ischemic stroke, and identifying the risk factors of AIS is crucial for figuring out the most effective preventive measures and treatment suggestions.^[11,12]^

At present, although ample studies have been carried out on AIS prediction on the basis of CTA or serum markers,^[13,14]^ there has been no research which combines multiple clinical data to establish a multivariate prediction model for AIS and use it in clinical management.

Therefore, the present paper reports a retrospective study combining demographic data, computer information technology and blood biomarkers. On the basis of 1 single risk factor screening, a joint prediction model is developed for the clinical diagnosis and treatment of AIS which is supposed to help clinicians formulate personalized treatment for patients and promote patient recovery.

## 2. Method

### 2.1. Study design

A single center retrospective clinical study was conducted in the Third People’s Hospital of Zigong City, Sichuan Province. The research team is comprised of members from the laboratory, radiology and neurology departments of the hospital.

### 2.2. Study registration

The TCTR identification number is TCTR20221014003.

### 2.3. Participants

Two hundred patients who met the inclusion and exclusion criteria were recruited through the hospital information system covering the period 2020 to 2022. The specific contents of the standard are given as follows:

### 2.4. Diagnostic criteria

Diagnostic criteria for AIS: Based on the 2015 American Heart Association/American Stroke Association Focused Update of the 2013 Guidelines for the Early Management of Patients With Acute Ischemic Stroke Regarding Endovascular Treatment: A Guideline for Healthcare Professionals From the American Heart Association/American Stroke Association (AHA/ASA),^[15]^ the 2018 Guidelines for the Early Management of Patients With Acute Ischemic Stroke, the 2019 Update to the 2018 Guidelines for the Early Management of Acute Ischemic Stroke,^[16]^ and the Chinese Guidelines for Endovascular Treatment of Acute Ischemic Stroke (2018).^[17]^

### 2.5. Inclusion criteria

Patients who have lived in the area for more than 1 year; Patients over 18 years of age; patients with available image data, clinical data and test index data.

### 2.6. Exclusion criteria

Patients with AIS history or with malignant tumors; Patients unwilling to cooperate or have mental disorders; Patients with missing clinical or follow-up information; Patients with low quality image.

## 3. Intervention

As this study is a retrospective analysis based on CTA images and blood biomarkers, no intervention measures were taken for patients.

## 4. Observation indicators and outcome

**Figure 1.**
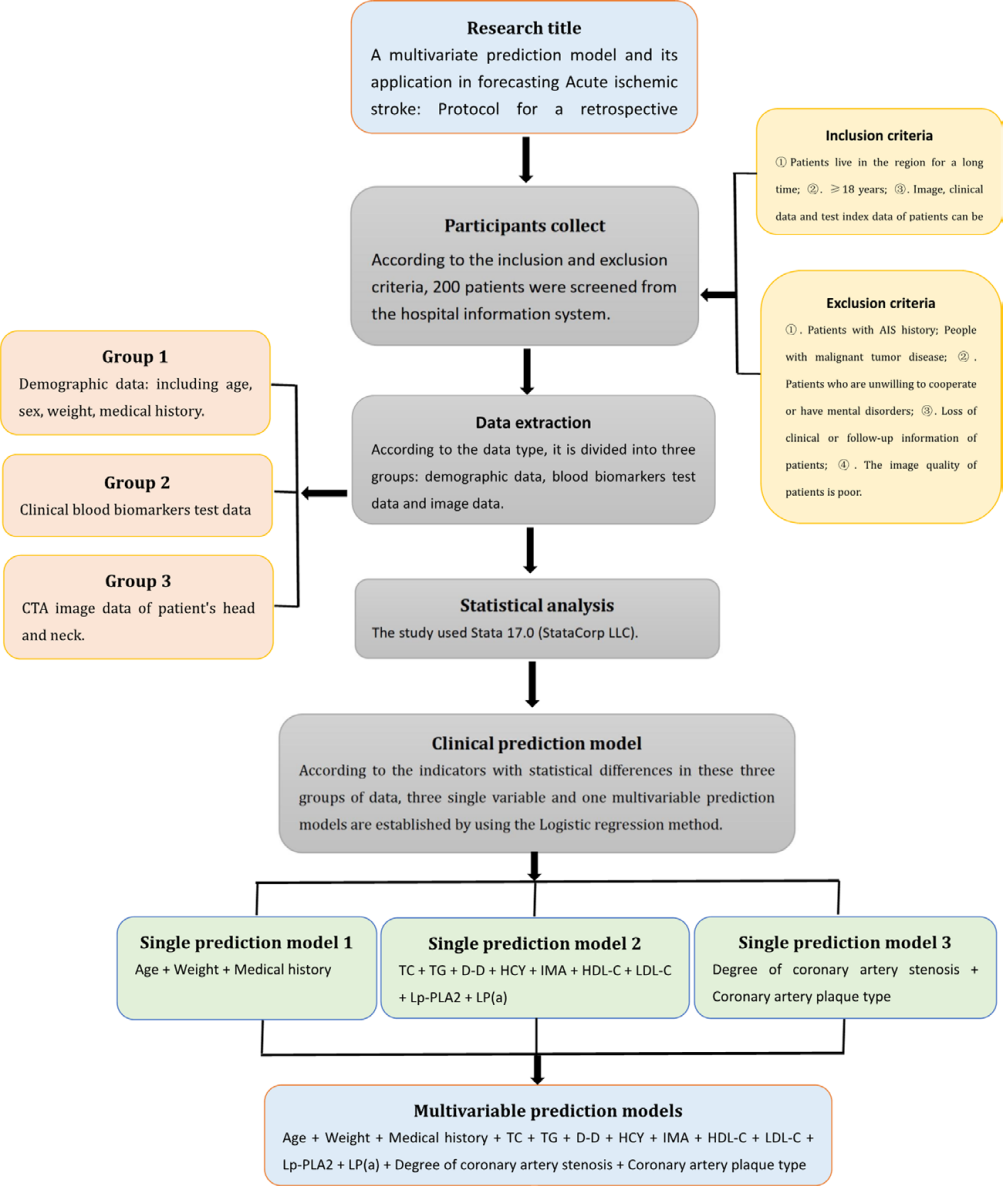
Flow chart of a multivariate prediction model and its application in forecasting Acute ischemic stroke: Protocol for a retrospective clinical study.

The clinical information of patients, including demographic data, clinical blood biomarker detection data and image data was collected, and logistic regression was used to establish 3 single factor prediction models and a multivariate prediction model. The prediction accuracy of the multivariate prediction model was evaluated by plotting the receiver operating characteristic (ROC) curve and comparing area under curve of the model (Fig. 1).

### 4.1. Demographic data

Demographic data included age, gender, body weight, and medical history, etc.

### 4.2. Blood biomarkers

Clinical blood biomarker testing data comprised D-Dimer (D-D); total cholesterol (TC), total triglyceride (TG), high density lipoprotein cholesterol (HDL-C), low density lipoprotein cholesterol (LDL-C), homocysteine (HCY), lipoprotein associated photosynthesis A2 (Lp-PLA2), ischemia-modified albumin (IMA), lipoprotein a (Lp [a]) and other indicators.

### 4.3. Image data

CTA scanning of the head and neck was performed for the patients, the required images were uploaded to the processing workstation, and image reconstruction was performed to record the degree of coronary stenosis and coronary plaque types.

### 4.4. Establishment of prediction model

Logistic regression was used to establish the models. Based on the analysis of the 3 indicators of age, body weight and medical history included in demographic data, prediction model 1 was established; by analyzing 9 blood indicators (i.e., D-D, TC, TG, HDL-C, LDL-C, HCY, Lp-PLA2, IMA, Lp [a]), prediction model 2 was developed and from the analysis of the 2 indicators of coronary artery stenosis degree and coronary artery plaque type recorded in CTA image data, prediction model 3 was established. Then, a multivariate prediction model was established by combining the 14 indicators included in the 3 groups of data.

Finally, to interpret the results of regression analysis, area under curve of the ROC curve was used to evaluate the predictive ability of different models on the outcome indicators, and DeLong Method analysis was used to compare the area under the ROC curve. *P* < .05 suggests a statistically significant test result.

## 5. Data collection and management

Any information that may reveal the participants’ personal identity, including name, and ID number, etc. was screened out, and strict security and confidentiality measures were formulated.

The research team held a special clinical training meeting and trained all the members concerning the implementation plan and standard operating procedures (SOP), so that each member of the research team was sure to be familiar with the procedure and specific operating methods of the study, so as to guarantee the consistency and reliability of the clinical study. The investigator statement was signed, and the SOP for observation and quality control of various indicators in the hospital laboratory were formulated. The team designated special personnel to be responsible for the quality control of the research so as to eliminate deviations in the research results.

**Figure 2.**
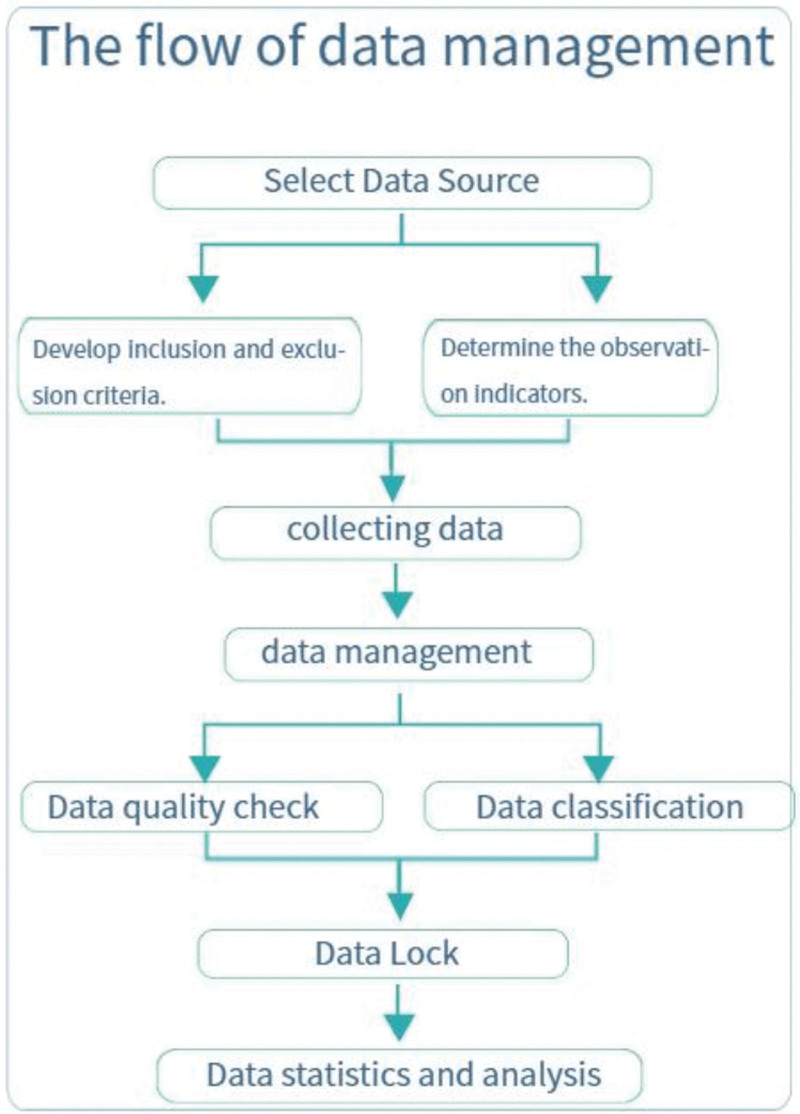
Flow chart of data collection and management.

The flow of data management is illustrated in Figure 2.

## 6. Statistical analysis

In this study, Stata 17.0 software was used to analyze the collected data. Before performing statistical analysis, we invited statistical analysts to make plans and specify detailed steps, then carefully examine the data collected. All statistical analysis processes were programmed and archived for future reference.

## 8. Discussion

In clinical applications, Liu Yubo^[18]^ analyzed the application value of carotid artery stenosis for early diagnosis of acute ischemic stroke on the basis of CTA technology. Through detection and analysis, it is known that TC, TG, LDL-C, hs CRP, HCY, Lp-PLA2, IMA, complement Clq, Lp (a) are the pathogenic factors of acute ischemic stroke.^[19,20]^ This study combined clinical information and medical imaging technology to establish a multivariate prediction model for the diagnosis of AIS, which provides suggestions for clinicians to design personalized treatment for patients, thus facilitating patient recovery.

There are several limitations in our research. For 1 thing, since this investigation is a single center retrospective study, more multicenter studies are needed in the future to verify the prediction effectiveness of the multivariate model. In addition, clinical research has variability, which may produce some impact of the research results. We need to improve the implementation plan according to the specific situation.

We hope that the multivariate prediction model can more accurately predict the occurrence of AIS, which has important clinical significance in the diagnosis of AIS.

## Author contributions

**Conceptualization:** Dongmei Yang, Li Wang, Jielian Li.

**Data curation:** Xia Liu, Hui Lan, Yu Xie.

**Methodology:** Dongmei Yang, Hui Lan, Xiao Ma.

**Resources:** Li Wang, Dongmei Yang, Jielian Li.

**Radiomics analysis:** Li Wang.

**Supervision:** Li Wang, Jielian Li.

**Writing - original draft:** Dongmei Yang, Xia Liu.

**Writing - review & editing:** Dongmei Yang, Li Wang, Jielian Li.
